# Efficacy of a Nickel-Titanium Ultrasonic Instrument for Biofilm Removal in a Simulated Complex Root Canal

**DOI:** 10.3390/ma13214914

**Published:** 2020-10-31

**Authors:** Young-Ryul Oh, Hye-Min Ku, Dohyun Kim, Su-Jung Shin, Il-Young Jung

**Affiliations:** 1 Department of Conservative Dentistry and Oral Science Research Center, Microscope Center, Yonsei University College of Dentistry, Seoul 03722, Korea; apokalips88@naver.com (Y.-R.O.); DOHYUN0KIM@yuhs.ac (D.K.); 2 Department of Preventive Dentistry & Public Oral Health, BK 21 PLUS Project, Yonsei University College of Dentistry, Seoul 03722, Korea; khm3193@gmail.com; 3 Department of Conservative Dentistry, Gangnam Severance Dental Hospital, Yonsei University College of Dentistry, Seoul 06273, Korea; SUJUNGSHIN@yuhs.ac

**Keywords:** nickel titanium, polydimethylsiloxane, ultrasonic, root canal, biofilm

## Abstract

This study evaluated the effectiveness of NiTi ultrasonic tips for *Enterococcus faecalis* (*E. faecalis*) biofilm removal in simulated complex root canals. Sixty root canal models consisting of a 30-degree curved main canal and two lateral canals were constructed from polydimethylsiloxane and incubated with *E. faecalis*. Irrigants in root canals were activated using a manual syringe (SI), a stainless steel (SS) instrument, a nickel-titanium (Ni-Ti) ultrasonic instrument, or a sonic instrument (EA). Instruments of SI, SS, and NiTi-9 groups were placed 9 mm from the apex, whereas those in NiTi-2 and EA groups were placed 2 mm from the apex. The efficacy of each method was determined as the ratio of fluorescence concentration before and after activation. In the apical curved canal, the highest efficacy was found in the NiTi-2 group (99.40%), followed by SI (84.25%), EA (80.38%), SS (76.93%), and NiTi-9 (67.29%) groups. In lateral canals 1 and 2, the efficacy was the highest in the NiTi-2 group and the lowest in the SI group. The NiTi ultrasonic instrument could effectively remove biofilms in the curved canal and lateral canals. This instrument should be introduced close to the working length. An up-and-down motion of the activation instrument is recommended.

## 1. Introduction

Disinfection of the root canal system is essential for successful endodontic treatment. However, disinfection is limited when using mechanical instrumentation alone because of anatomic complexities in root canal systems, such as curvatures, fins, grooves, isthmus, and lateral canals [1,2]. There is an increasing awareness of the importance of antimicrobial irrigants when cleaning these systems.

Sodium hypochlorite (NaOCl) is a commonly used irrigating solution with a broad spectrum of antimicrobial actions and has the ability to dissolve both necrotic and vital pulp tissues [3]. A recent study reported that irrigants affect operative torque during root canal instrumentation [4]. The operative torque means that the amount of torque needed by the rotary instrument to reach the root apex, and NaOCl reduces operative torque during the treatment. However, a syringe irrigation method with NaOCl has some limitations, including the stagnation of the irrigant and vapor lock [5,6]. Various activation methods have been used to increase the penetration of irrigants and improve their efficacy. Sonic and ultrasonic devices are typically used [7]. The energy from the ultrasonic device is transmitted from an oscillating wire and file to the irrigant through ultrasonic waves that induce two physical phenomena: acoustic streaming and cavitation of the irrigant [8,9,10,11]. Acoustic streaming is defined as a rapid movement of the fluid in a circular or vortex-like shape around the vibrating file. In contrast, cavitation is defined as the generation of steam bubbles [8]. The acoustic streaming promoted by the ultrasonic device disrupts bacterial aggregation [12]. Passive ultrasonic irrigation produces higher frequency oscillation than sonic irrigation, ranging between 25 and 40 kHz [13]. On the other hand, sonic activation generates mechanical oscillation at the irrigation tip. Sonic activation is performed with flexible polymer tips to prevent cutting the root canal wall [14].

Many investigators have suggested that root canals are significantly cleaner after sonic or ultrasonic activation than after irrigation with a manual syringe [2,15,16,17]. However, these activation methods also have some limitations. When treating the apical portion of curved main canals, unintended contact between the ultrasonic file and the root canal wall is inevitable because of the root canal system’s dimensions and complex geometry [18,19]. This contact can reduce the ultrasonic energy and increase the risk of iatrogenic cutting of the root canal wall and instrument separation [19].

In the sonically activated tip, the apical root canal’s small diameter can inhibit free oscillation of the tip, reducing the stream of irrigants into the canal, preventing cavitation either on the sonic tip or on the canal wall [14]. There are some controversies on whether sonic or ultrasonic irrigation is more effective [13,20,21]. Previous studies on the efficacy of root canal irrigation have used extracted teeth (single or multiple-rooted) or oversimplified root canal models [3,18,21,22]. The advantage of using extracted teeth is that it is easy to perform an experiment that reproduces a clinical situation. When evaluating the efficacy of various irrigation methods, the standardization of canals is of utmost importance. However, extracted teeth have very different morphological features, making it difficult to use them in in vitro studies under standardized conditions [23]. To overcome these limitations, a previous study [24] attempted to make artificial root canal models using transparent resin material and three-dimensional (3D) printing to visualize the experimental disinfection of a root canal. Thus, we created a transparent, standardized, and anatomically complex root canal model that included the root canal curvature and lateral canals.

*Enterococcus faecalis* (*E. faecalis*) was the most frequently used test organism in endodontic biofilm model systems because it is frequently isolated from root canal-treated teeth with persistent apical pathosis [25]. *E. faecalis* was previously detected in approximately 18% of primary endodontic infections. In approximately 67% of endodontic failure cases [26], *E. faecalis* infection was considered to be the main cause of endodontic treatment failures [27].

A nickel-titanium (Ni-Ti) ultrasonic (Endosonic Blue, Maruchi, Chuncheon-si, Korea) file has been recently introduced into the market by Maruchi Co., Ltd. (Wonju, Korea). The Ni-Ti file reportedly tends to straighten within a curved canal [28], resulting in an unintentional cut in the canal wall. However, the Ni-Ti instruments in this study were in R-phase, which has high elasticity, enables pre-bending, and improves cyclic fatigue resistance. This minimizes the risk of iatrogenic cutting of the canal wall and enhances fundamental flexibility.

The aim of this study was to compare the efficacy of different activation methods for removing *E. faecalis* biofilm in a transparent root canal model. The irrigation efficacy based on location was also evaluated.

## 2. Materials and Methods

### 2.1. Fabrication of Root Canal Models

Root canal models were simulated on microfluidic chips made with polydimethylsiloxane (PDMS) using a soft lithography technique [29]. A coat of negative photoresist (SU-8; MicroChem Inc., Westborough, MA, USA), which becomes insoluble when exposed to light, was applied to a silicon wafer and exposed to 365 nm ultraviolet light through a mask to make a mold for a channel. PDMS was then cast in the mold and bonded on a pre-fabricated flat PDMS block to make a microfluidic chip with an internal channel.

**Figure 1:**
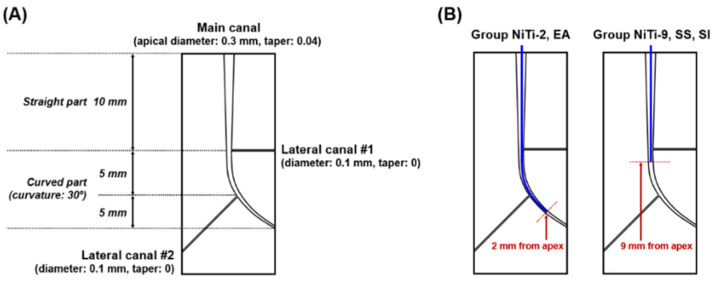
Schematic design of the simulated root canal model, including the lateral canal and the curved canal’s apical portion. The lateral canals have a diameter of 0.1 mm. The curved canal’s apical portion is created with an angle of 30 degrees from the straight main canal (**A**). The image on the right shows the five irrigation methods used in this study. Each method is indicated by a colored line in the designed root canal, representing the placement of irrigation instruments in this simulated anatomical structure (**B**).

The microfluidic chip had a cuboid shape (20 mm × 40 mm × 5 mm). The simulated root canal consisted of a curved main canal and two lateral canals (Figure 1).

The main canal consisted of a single curved canal with an apical size of #30 (0.3-mm diameter) and a 0.04 taper, which means that the diameter increases by 0.04 mm per 1 mm from the apex. A 30-degree curvature was made about 10 mm from the apex according to the Schneider method [30]. Two lateral canals, each with a diameter of 100 µm, ramified from the main canal at 5 mm and 10 mm from the apex. The root canal model used in the study was a closed system.

### 2.2. Formation of E. faecalis Biofilm within the Root Canal Model

*E. faecalis* (ATCC 19433) was inoculated into brain heart infusion (BHI) broth (Difco Laboratories Inc., Detroit, MI, USA) at a concentration of 1 × 10 CFU/mL and then incubated at 3 °C for 24 h with 80% N_2_, 10% CO_2_, and 10% H_2_. Pure *E. faecalis* cultures were confirmed by colony morphology on BHI agar plates before the biofilm was generated. After 24 h of incubation, the root canal models were inoculated with 200 µL of *E. faecalis* for biofilm formation. The BHI broth was replenished daily and kept in an incubator for 10 days for the biofilm to develop.

The biofilm was visualized and quantified by a crystal violet binding assay, as previously described [22]. After 10 days of incubation with *E. faecalis*, loosely adherent bacteria inside the simulated root canal were rinsed out with 1 mL of sterile distilled water. The remaining biofilm was stained with 1 μL of crystal violet (212525; Becton, Dickinson and Company, Franklin Lakes, NJ, USA) for 1 min. Stained canals were subsequently washed with 3 mL of sterile distilled water for 1 min. The root canal model was placed on a microscopic slide table and observed under an Axio Imager M2 fluorescence microscope (Carl-Zeiss, Oberkochen, Germany). Images of two lateral canals and the apical portion of the main canal were captured (at 5× magnification) and analyzed with a ZEN pro software (Carl-Zeiss, Oberkochen, Germany). The exit portals of the lateral and main canals were then blocked with sticky wax.

### 2.3. Irrigation Procedures

Sixty root canal models were fabricated and randomly divided into five experimental groups (five different activation methods), as shown below. In all groups, a total of 9 mL of 3% NaOCl was delivered using a 10 mL syringe with a beveled 27-G needle. The needle was placed 9 mm from the apex into the canal. The syringe was attached to a programmed syringe pump to deliver the irrigant at a constant flow rate of 5 mL/min.

#### 2.3.1. SI Group (Control)

In the syringe irrigation (SI) group, after the irrigation described above, the irrigant was left stagnant in the root canal model for 30 s. Then, all the debridement was washed out using 0.3 mL of 3% NaOCl.

#### 2.3.2. SS Group

In the stainless steel (SS) group, the agitation was carried out using a stiff stainless steel instrument (DH tip; Epdent Co., Ltd., Seoul, Korea), which was mounted on a Mini Piezon ultrasonic power unit (EMS Electro Medical Systems SA, Nyon, Switzerland). The instrument’s tip was placed 9 mm from the apex into the canal, which was 1 mm short of its binding point in the curved canal. It was then activated at a power level of 4 with an ultrasonic power unit for 10 s, as directed by the manufacturer. Then, 0.1 mL of 3% NaOCl was used for the washout debridement. The activation and debridement procedure described above was repeated two more times.

#### 2.3.3. NiTi-9 Group

The entire process was the same as that for the SS group except a #15/.02 NiTi ultrasonic instrument (Endosonic Blue, Maruchi, Chuncheon-si, Korea) was used instead of the SS one.

#### 2.3.4. NiTi-2 Group

The same NiTi instrument was placed 2 mm from the apex into the curved canal. The entire process was the same as that used for the NiTi-9 group.

#### 2.3.5. EA Group

In the endoactivator (EA) group, agitation was carried out using an Endoactivator (Endoactivator, Dentsply Tulsa Dental, Tulsa, OK, USA) device, instead of an ultrasonic power unit, by placing the polymer tip with size #15/.02 2 mm from the apex into the curved canal. Then, activation was performed in a cyclic axial motion at the highest speed for 30 s, as suggested by the manufacturer. This was followed by debridement using 0.3 mL of 3% NaOCl.

After activation of the irrigant, crystal violet staining was repeated, and the same area captured before irrigation was recaptured with the same microscope to measure the amount of remaining biofilm remaining.

### 2.4. Effects of Irrigation on Removal of E. faecalis Biofilm

**Figure 2:**
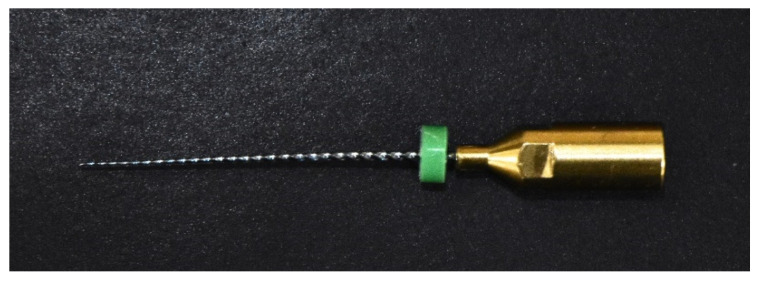
Ni-Ti ultrasonic instrument.

For each root canal model, images of two lateral canals close to the main canal and the area 2 mm from the apex were captured at 5× magnification with a fluorescence microscope before and after irrigation. All images were obtained from one side. The biofilm’s fluorescence concentration was measured for each image (Figure 2) and analyzed using an Image-Pro Plus 6.0 software (Media Cybernetics, Inc., Washington, DC, USA).

The efficacy of irrigation was determined as the ratio of fluorescence concentration before irrigation to that after irrigation. To obtain images of the same location before and after irrigation, the same investigator marked a capturing point on the canal model’s surface. The point where the lateral canal branched off from the main canal was used as the starting point of the image. The investigator marked the endpoint on the canal model to maintain the same capturing location of the lateral canal in all models. All images captured the same area three times with intervals, and their average values were used for analysis.

### 2.5. Statistical Analysis

Normality of data was confirmed by the Shapiro-Wilk test. A linear mixed model was used to compare the efficacy of irrigation between the study groups. Differences in efficacy among the five irrigation methods depending on canal location were analyzed as method × location interactions. Tukey’s post-hoc analyses were used to compare differences in the efficacy of irrigation between different methods and locations. All statistical analyses were performed using SAS version 9.3 (SAS Institute Inc., Cary, NC, USA). A *p*-value <0.05 was considered statistically significant.

## 3. Results

**Figure 3:**
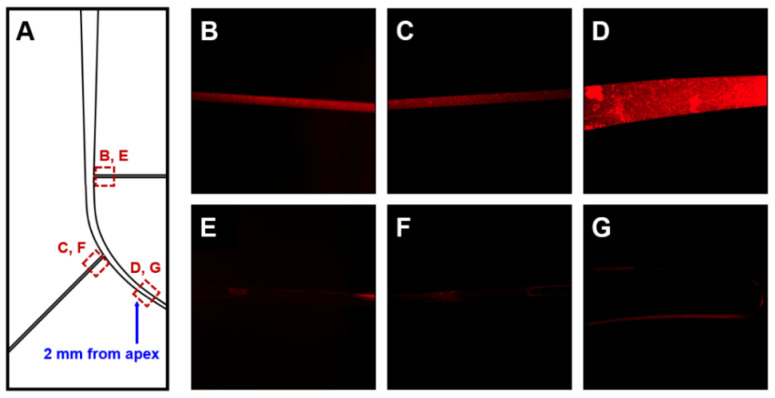
Fluorescence microscope images of *Enterococcus faecalis* biofilm at each root canal location (5× magnification) (**A**). The upper images show the growth of biofilm after incubation in lateral canal 1 (**B**), lateral canal 2 (**C**), and the apical portion of the curved canal (**D**). The lower images show each canal image after removal of biofilm from lateral canal 1 (**E**), lateral canal 2 (**F**), and the apical portion of the curved canal (**G**) after irrigation.

Results for efficacy of irrigation according to each location in root canal model and irrigation methods are shown in Table 1 and Figure 3.

The linear mixed model analysis showed a significant difference in irrigation efficacy between methods according to canal location (*p* = 0.0195, Table 1). When the irrigation method was fixed, the efficacy of irrigation differed significantly according to root canal location (*p* = 0.0018; method, fixed effect). When the root canal location had a fixed effect, there was a significant difference in the efficacy of irrigation according to the irrigation method (*p* = 0.0001, fixed effect: canal location).

**Figure 4:**
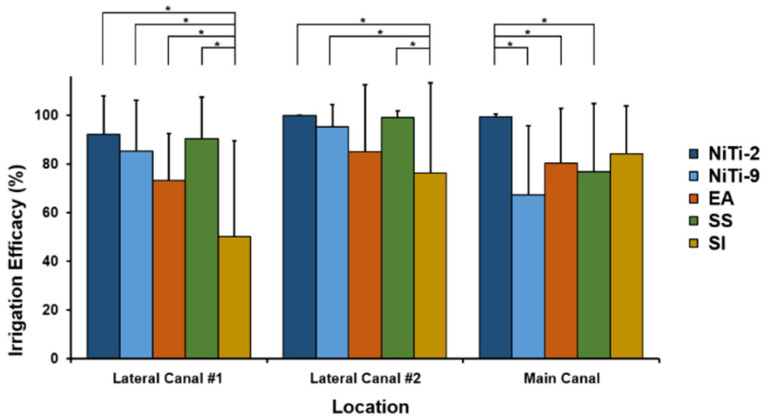
Irrigation efficacy of different activation methods at each location in the root canal. For NiTi-2, an ultrasonic irrigation instrument is placed 2 mm from the apex; for NiTi-9, an ultrasonic irrigation instrument is placed 9 mm from the apex; for endoactivator (EA), a sonic activator instrument is placed 2 mm from the apex; and for stainless steel (SS), ultrasonic irrigation is performed with a stainless steel instrument placed 9 mm from the apex. SI indicates syringe irrigation. Post-hoc analysis found a significant difference between the different methods (*p* < 0.05), represented by an asterisk.

For the apical area of the main canal, the NiTi-2 group showed the highest irrigation efficacy (99.40%), followed by SI (84.25%), EA (80.38%), SS (76.93%), and NiTi-9 (67.29%) groups (Figure 4).

The irrigation efficacy was significantly higher in the NiTi-2 group than in the NiTi-9, SS, or EA group. For lateral canal 1, the irrigation efficacy was the highest in the NiTi-2 group (92.10%) and the lowest in the SI group (50.04%; Figure 3, Table 1). Irrigation efficacy was significantly lower in the SI group than in any other study group. For lateral canal 2, the irrigation efficacy was highest in the NiTi-2 group (99.86%) and lowest in the SI group (76.19%). The efficacy was significantly lower in the SI group than in the NiTi-2, NiTi-9, or SS group. All methods showed better efficacy for lateral canal 2 than lateral canal 1, although such findings were significant only for the SI group.

## 4. Discussion

In this study, we evaluated NiTi ultrasonic tips’ effectiveness in a simulated curved root canal model made with a PDMS microfluidic chip.

The NiTi-2 instrument showed the best performance for biofilm removal in the apical area of the canal. The efficacy of NiTi-2 was significantly better than that of other activation methods but not higher than SI. The biofilm removal was less effective in the EA group than in the NiTi-2 group, meaning that the ultrasonic activation method was more effective than the sonic activation using an Endoactivator device; this finding is consistent with previous studies [13,14,31]. The difference in efficacy might be due to differences in driving frequency, which was 30 kHz for the ultrasonic device and 160–190 Hz for the sonic device. The ultrasonic device’s higher frequency could induce a faster irrigant flow, resulting in effective acoustic streaming. Transient cavitation generated by ultrasonic devices might have also contributed to such a difference. By contrast, a sonic device generates mechanical oscillation at the irrigation tip without cavitation [13]. It is believed that only the mechanical oscillation in a small curved canal is limited, in terms of increasing NaOCl’s efficacy.

When only ultrasonic devices were compared, there was no significant difference in efficacy between SS and NiTi-9 groups, and the NiTi-2 group had better efficacy than SS and NiTi-9 groups. These findings indicate that where the device is placed is more important than what it is made of. It can be inferred that the acoustic streaming and cavitation effect of the ultrasonic device are limited to the area close to the instrument tip. An SS ultrasonic file can be used in a curved canal when the file is precurved. However, accurate bending is difficult in a clinical situation, which may cause severe file–wall contact, resulting in a poor streaming effect [9,18]. Thus, we chose the NiTi ultrasonic file that could be used all the way to the working length easily.

Ultrasonic instruments tend to fracture during use. The main cause of the fracture appears to be cyclic fatigue due to continuous oscillation [32]. Therefore, the instrument should show improved cyclic fatigue resistance. Several kinds of heat-treated NiTi alloys are available to manufacture endodontic instruments, including M-wire, R-phase (rhombohedrally distorted martensite phase), and CM wire [28,33,34]. Previous studies reported that the R-phase instrument revealed superior cyclic fatigue resistance and flexibility compared to conventional NiTi without heat treatment. The NiTi ultrasonic instrument used in this study is made by R-phase heat treatment technology and benefited from pre-bending. This pre-bending is due to the R-phase’s elastic modulus being lower than that of other alloys [28,34]. Therefore, the R-phase instrument allows a more effective canal preparation and irrigation procedure than do conventional systems. These properties of the NiTi instrument with the R-phase are appropriate for use in the irrigation procedure in curved root canals. Additionally, the size of the ultrasonic instrument used in the present study is #15/.02. If the diameter of the file is larger than the width of the root canal, a small diameter file is recommended because the contact with the canal wall will interfere with the creation of free oscillation [35].

On the other hand, the efficacy was lower in the SI group than in the NiTi-2 group, but the difference between them was not significant. This means that 9 mL of irrigant with a 5 mL/min flow rate could also have adequate irrigation efficacy. It could be argued that the activation time and irrigant volume differed according to the method used in this study. Given that the optimal time and volume were not conclusive for each method, we did not consider that all the conditions should be the same for each method. Furthermore, this was not the aim of this study. Nonetheless, improved efficacy may be possible if a longer duration is used. However, increasing the treatment duration may be a disadvantage in clinical practice.

The results for lateral canals with a diameter of 100 µm were different from those for the main canal. All ultrasonic methods were significantly better than the SI group for both lateral canals. The findings of the EA group were better for the lateral canal 1 than in the SI group. These results indicate that activation of the irrigant is important in removing biofilm in lateral canals with a diameter of 100 µm, although the activation methods might not be perfect. This result is consistent with that of a previous study [36], confirming that it is difficult to remove biofilm formed in lateral canals with a small diameter, regardless of the irrigant used in traditional irrigation processes.

In all groups, more biofilm was removed from lateral canal 2 than from lateral canal 1, although the difference was only significant in the SI group. These findings suggest that the irrigation efficacy differed according to the instrument’s location, consistent with a previous study showing differences in cleaning efficiency [37]. The previous study suggested that the ultrasonic file’s effect would be more intense at the apical section of the file because of acoustic streaming. Therefore, an up-and-down motion of the instrument or syringe is useful when cleaning a root canal system with lateral canals.

The PDMS is used for biomedical applications, such as microreactors, microchips for capillary gel electrophoresis, hydrophobic vent valves, and soft lithography [38,39]. Bacterial invasion of dentinal tubules commonly occurs in endodontic infection [40]. Although a porous structure similar to the dentin surface could not be reproduced using the PDMS material, a recent study has shown that PDMS-based root canal model is suitable for the imaging analysis of irrigation methods because it is transparent and has a contact angle with NaOCl similar to that of dentin [41]. Song et al. reported that material properties affect bacteria–surface interaction and that the biofilm adheres to PDMS [42]. A previous study using PMDS for biofilm removal has demonstrated bacterial growth and adhesion on this material’s surface under conditions similar to those used for bacterial growth and adhesion to dentin [43,44]. Experimental conditions using a porous structure of dentin are recommended for further in vivo studies.

The mean primary curvatures of the mandibular first and second molars of the mesiobuccal canal have been reported to be 28.7 degrees [45]. Therefore, in this study, a simulated canal was fabricated with a curvature angle of 30 degrees from the end of the canal (Schneider method [30]), similar to a previous study [3], which was a strength of this investigation. Previous studies have evaluated the efficacy of irrigation to remove bacteria only in the main canal without considering the lateral canal [22,46]. Therefore, we developed a root canal model that included the lateral canals with a diameter of 100 µm and an anatomic structure similar to that of normal teeth [47]. To our knowledge, our study is the first to create a root canal model that includes both curvature and lateral root canals.

## 5. Conclusions

In all the root canal systems fabricated in the study with a curved main canal and two lateral canals, ultrasonic activation using a NiTi instrument improved biofilm removal. Because improved efficacy was found in the area around the instrument tip, clinicians should strive to make the tip come in contact with the root canal’s entire length. Therefore, an up-and-down motion of the instrument is useful when cleaning a root canal system with lateral canals.

## Figures and Tables

**Table 1:** The percentage of mean irrigation efficacy values in terms of removing biofilm before and after irrigation using different irrigation methods.

Location	Mean Irrigation Efficacy (SE)					*p*
	NiTi-2	NiTi-9	SS	EA	SI	
Lateral canal #1	92.10 (6.80)	85.36 (7.40)	90.38 (7.08)	73.28 (7.40)	50.04 (6.80)	Method: 0.0001 Location: 0.0018 Method*Location: 0.0195
Lateral canal #2	99.86 (5.93)	95.14 (6.44)	99.14 (6.17)	84.95 (6.44)	76.19 (5.93)	
Main canal apex	99.40 (6.06)	67.29 (6.58)	76.93 (6.30)	80.38 (6.58)	84.25 (6.06)	
A linear mixed model analysis of the effect of location and method on irrigation efficacy in each root canal according to the type of irrigation method used. NiTi, nickel titanium; SE, standard error						
